# Impact of Ecobiol plus ® feed additive on growth performance, physiological response, oxidative status and immunological status of Nile tilapia (*Oreochromis niloticus*) fingerlings challenged with *Aeromonas hydrophila*

**DOI:** 10.1186/s12917-025-04480-x

**Published:** 2025-01-31

**Authors:** Amira A. Omar, Doaa H. Assar, Mustafa Shukry, Aya Abo El-Ezz, Foad A. Farrag, Wesam E. Abd El-Aziz, Eman M. Moustafa

**Affiliations:** 1 Fish Diseases and Management Department, Faculty of Veterinary Medicine, Kafrelsheikh University, Kafrelsheikh, 33516 Egypt; 2 Clinical Pathology Department, Faculty of Veterinary Medicine, Kafrelsheikh University, Kafrelsheikh, 33516 Egypt; 3 Animal Physiology Department, Faculty of Veterinary Medicine, Kafrelsheikh University, Kafrelsheikh, 33516 Egypt; 4 Anatomy and Embryology Department, Faculty of Veterinary Medicine, Kafrelsheikh University, Kafrelsheikh h h, Kafr El-Shaikh, 33516 Egypt

**Keywords:** Aquaculture, Probiotic supplementation, Fish immunity, Oxidative stress, Nile tilapia nutrition, *Aeromonas hydrophila* challenge

## Abstract

This research investigates the effects of dietary supplementation with Ecobiol plus^®^ (Bacillus amyloliquefaciens CECT5940) on the growth performance, physiological responses, oxidative stress, and immune status of Nile tilapia (Oreochromis niloticus) fingerlings revealed to *Aeromonas hydrophila*. A total of 525 Nile tilapia fingerlings, averaging 30.00 ± 5.00 g in initial weight, were randomly divided into four groups (control and three experimental groups), each with three replicates of 25 fish. Ecobiol plus^®^ was integrated in the diet at concentrations of 0.0 (control), 0.1, 0.2, and 0.4 g/kg feed. Fish were fed at 3% of their biomass daily, with biweekly adjustments based on growth. The feeding trial lasted 8 weeks, followed by a 15-day challenge with *Aeromonas hydrophila* to evaluate immune responses and survival outcomes. The group receiving 0.4 g/kg of Ecobiol plus^®^ exhibited the most significant improvements in growth performance, including higher weight gain, specific growth rate, and feed conversion efficiency (*p* < 0.05). Blood and biochemical assessments indicated increased hemoglobin, total protein, and globulin levels, reflecting improved physiological and immunological conditions. Additionally, lysozyme activity and phagocytic response were markedly enhanced, demonstrating the probiotic’s immune-boosting potential. Histopathological evaluations revealed reduced gut, gills, and hepatopancreas lesions, especially in groups with higher supplementation levels. Fish in Group T4, fed with 0.4 g/kg Ecobiol plus^®^, achieved the best growth performance, with a final weight of 77.96 ± 5.53 g, a weight gain of 42.46 ± 3.18 g, and a specific growth rate (SGR) of 1.51%/day, compared to the control group (T1) with a final weight of 75.26 ± 3.87 g, weight gain of 35.76 ± 2.08 g, and SGR of 1.24%/day. Survival rates considerably increased in the treated groups, with T4 exhibiting the highest rate of 96.00%, followed by T2 (94.68%), T3 (92.00%), and T1 (81.32%). Additionally, the LD50 of *A. hydrophila* was determined to be 3 × 10⁷ CFU/mL, underscoring the protective effect of Ecobiol plus^®^ in enhancing fish immunity and resilience against bacterial challenges. These results suggest that Ecobiol plus^®^ can be a natural, antibiotic-free additive to strengthen growth and disease resistance in Nile tilapia. The optimal inclusion level of Ecobiol plus^®^ is 0.4 g/kg for maximum benefit.

**Supplementary Information:**

The online version contains supplementary material available at 10.1186/s12917-025-04480-x.

## Introduction

Aquaculture is vital in ensuring global food security, providing a substantial proportion of animal protein for human consumption. Nile tilapia (Oreochromis niloticus) is essential worldwide due to its quick growth, adaptation to different environments, and cost-effectiveness for production. However, intensified aquaculture practices have increased disease outbreaks, primarily bacterial infections, compromising production and sustainability [1, 2]. Aquaculture is expanding rapidly to supply humans with a substantial quantity of animal proteins [3]. Aquaculture constitutes Egypt’s predominant source of fish production [4, 5]. Consequently, aquaculture practices have been intensified to maintain high production levels, increasing stress on aquatic animals and the environment [6]. Excessive antibiotic usage to control these infections has raised serious issues about antimicrobial resistance and environmental effects, necessitating the development of natural and sustainable alternatives [7].

Nevertheless, the applications of these substances led to many complications, and their perils might indirectly impact human well-being. Therefore, creating growth stimulants and natural substitutes has become urgently necessary for aquatic animal health [8–10]. The marine animals’ health is significantly influenced by the host terrestrial microorganisms, which perform vital functions such as stimulating the immune system, helping digest nutrients, and enhancing resistance to sophisticated diseases [11]. The functional integrity of intestinal microbiota, which has the potential to influence biological processes for the betterment of the host, is significantly reliant on the microbes’ capacity to interface with the digestive tract [8, 12].

Aquaculture operations rely heavily on fish feed as an expensive input due to the critical nature of fish nutrition in regulating the health of farmed shellfish and finfish [13]. A food item or condiment can be “fortified” by adding micronutrients (such as vitamins and minerals) to increase nutritional value and decrease health hazards. In addition to containing micronutrients lost during processing, micronutrients can improve the nutritional value of staple foods [14–16].

The need for more ecologically responsible aquaculture has increased research into probiotics as a possible therapy for aquatic creatures. Probiotics are dietary supplements containing live microorganisms that enhance the health of terrestrial livestock and humans [17–19]. Incorporating probiotics in aquatic organisms’ nutrition can benefit their physiological conditions, growth performance, and feed utilization [1, 20]. Nutritional probiotics can be assimilated optimally to bolster the immune system, particularly during stress. There are specific circumstances in which additional probiotics are required to stimulate immune cells further [21, 22].

Probiotics have gained attention as a promising solution to these challenges, offering enhanced growth, improved immune responses, and better gut health [23, 24]. Aly et al. [25] recorded that *O. niloticus* grew more when *Bacillus pumilus* was added as a feed additive for two months. Furthermore, tilapia immunity, health, and growth were all enhanced by adding *B. coagulans* B16 to water [26].

Among these, *Bacillus amyloliquefaciens* has shown significant potential as a probiotic in aquaculture, yet there is a dearth of studies on its specific effects on Nile tilapia under pathogenic challenges. Feeding *B. amyloliquefaciens* enhanced disease resistance, increased immunological activity in channel catfish, catla, and eel, and superior development and a better (FCR) in Nile tilapia [27]. Even so, no extensive research has ever been done on how *B. amyloliquefaciens* supplementation affects *O. niloticus* growth, intestinal morphometry, immunity, and overall health. Ecobiol Plus ® is a probiotic composed of *B. amyloliquefaciens* CECT5940 spores, which contributes to maintaining a healthy gut microflora (1 × 10 ^6^ CFU).

This study aims to address the existing research gaps, including the lack of detailed knowledge about the impact of *Bacillus amyloliquefaciens* on physiological, immunological, and oxidative responses in Nile tilapia and the optimal dosage of Ecobiol plus^®^ for achieving maximum health benefits. This study hypothesizes that including Ecobiol plus^®^ in the diet of Nile tilapia promotes improved growth performance, boosts immune functionality, and increases disease resistance, especially during exposure to *A. hydrophila*. The primary objectives are to examine its effects on growth parameters, blood-related and metabolic indicators, survival rates, and tissue responses while determining the most effective dosage for practical aquaculture applications.

## Materials and methods

### Ethical approval

The ethics criteria set out by the Kafrelsheikh University Ethics Committee in Egypt (KFS-IACUC/138/2023) for the use and care of animals were meticulously followed throughout all processes involving fish alteration.

### Experimental diet

Minerals, vitamins, fish oil, wheat bran, soybean meal, yellow maize, fish meal, and other isonitrogenous and isocaloric components comprise the base (control) diet. Digestible energy content was 12.6 MJ/kg, and crude protein content was 30%. These were sourced from the ALEKHWA^®^ feed factory in Kafr El-Sheikh, Egypt (Table 1). A feed processor crushes the dry ingredients into tiny pieces. In the 2nd, 3rd, and 4th diets, As a filler, rice bran was utilized, and the probiotics (Ecobiol plus^®^; Evonic spana y Portugal S.A.) were incorporated at different concentrations: 0, 0.1, 0.2, 0.4 gm/kg ration to the baseline diet). Ecobiol plus^®^ is a commercially available probiotic product containing *Bacillus amyloliquefaciens* CECT5940 spores at 1 × 10⁶ CFU/g, along with inert carriers such as calcium carbonate. This product enhances gut microbial balance and immune response in aquaculture species. Diets were designed to be isonitrogenous and isocaloric so that nutritional profiles would be constant throughout treatments. After thoroughly combining them, the components included (water, oil, vitamins, mineral mixture, and dicalcium phosphate). A combination blender weighed and combined the contents for 20 min. As suggested by Shimeino et al. [28], each 100 gm/diet was added to the blend progressively following homogeneous blending. Pelletizing the dough was done using a 2–3 mm die. We air-dried the pellets and then kept them in a four-degree fridge. The feed additive, Ecobiol plus^®^ (*Bacillus amyloliquefaciens* CECT5940, 1 × 10⁶ CFU/g), represents the spore concentration in the product, not in the gut. Produced by Evonic Spana y Portugal S.A., its nutritional profile was verified using standardized methods [29]. The filler in this recipe was rice bran to maintain the isonitrogenous and isocaloric nature of the diet. Including Ecobiol plus^®^ at different concentrations (0.1, 0.2, and 0.4 g/kg) required slight reductions in rice bran content to accommodate the additive while ensuring the overall nutrient profile of the diet remained balanced. The reduction of rice bran did not equate to the supplementation amount of Ecobiol plus^®^ due to the concentrated nature of the probiotic.

**Table 1:** Composition and chemical analysis of experimental diets (on dry matter basis)

Components	Diets			
	T1	T2	T3	T4
Fish meal (72% CP)	110	110	110	110
Soybean meal (45% CP)	360	360	360	360
Wheat bran	200	200	200	200
Yellow corn	60	60	60	60
Rice bran	200	200	199	198
Ecobiol plus^®^	0	0.1	0.2	0.4
Fish oil	15	15	15	15
Soybean oil	15	15	15	15
Dicalcium phosphate	10	10	10	10
Vitamins mixture	10	10	10	10
Minerals mixture	10	10	10	10
Carboxymethyl cellulose	10	10	10	10
Total	1000	1000	1000	1000
Chemical analysis				
Dry matter	91.5	91.3	91.8	91.2
Crude protein	31.3	31.3	31.3	31.3
Ether extract	8.1	8.1	8.1	8.1
Total ash	7.33	7.37	7.3	7.31
Crude fiber	6.6	6.68	6.65	6.62
Nitrogen-free extract	46.7	46.4	46.7	46.5
Gross energy (kcal/g)^1^	4.71	4.72	4.74	4.7

^1^Gross energy (GE) was calculated from NRC [59] as 5.65, 9.45, and 4.11 kcal/g for protein, lipid, and carbohydrates, respectively. T1= (control group), T2= (0.1 g/kg Ecobiol plus^®^), T3= (0.2 g/kg Ecobiol plus^®^), T4= (0.4 g/kg Ecobiol plus^®^),

### Experimental plan

Ecobiol plus^®^ (Bacillus amyloliquefaciens CECT5940, 1 × 10⁶ exceed CFU/g) was used as the feed additive in this study, with the spore concentration referring to the product rather than the gut microbiota. Manufactured by Evonic Spana y Portugal S.A., the nutritional content of the test diets was confirmed through standardized analysis.

After the initial acclimation period, the fish fingerlings were divided into four equal groups, each receiving three copies and each replica containing twenty-five fingerlings. Aeration was efficient, and 25 fish per aquarium were housed in 60 × 35 × 40 cm glass tanks. The fingerlings weighed 30.00 ± 5.00 g. Seventy liters of water were also present in the aquariums, resulting in a stocking density of 1.07 kg per 70 L. Water quality was maintained through continuous aeration and 50% daily water exchanges, ensuring optimal dissolved oxygen levels, pH, and nitrogenous wastes. The fish displayed normal behavior throughout the experiment, with no signs of stress or aggression, indicating the suitability of the setup for the study’s objectives. Meals supplemented with 0.1, 0.2, and 0.4 gm/kg ration of Ecobiol plus^®^ were provided to Groups 2–4, whereas Group 1 (control group, T1) received commercial basal diets. T2, T3, and T4, in that order. Fish were fed the recommended diet for eight weeks, which amounted to 3% of the total biomass in the tank. At the beginning of the research phase, we weighed the fish every two weeks; we adjusted the meal amounts based on the fluctuations in their live body weight. Each tank’s water level was roughly doubled daily by adding dechlorinated fresh water, and fish and feeding waste were removed via siphoning.

The American Public Health Association reports that water analysis instruments (Lamotte device, USA) were used to examine water parameters twice weekly [30]. Throughout the trial, the environmental parameters were maintained as follows: temperature ranged from 24 to 27 °C, dissolved oxygen was 6.5 ± 0.5 mg/L, pH was 7.1 ± 0.8, electrical conductivity (EC) was 219 ± 2 µmho/cm, total ammonia was kept below 0.1 mg/L, and a 12:12-hour light-dark photoperiod was implemented (Table 2).

**Table 2:** Water Quality parameters measured in the current study

Parameters	T1	T2	T3	T4
Salinity (ppt)	10	10	10	10
Temperature (◦C)	24.3 ± 0.32^b^	25.9 ± 0.09^a^	26.3 ± 0.03^a^	26.9 ± 0.02^a^
pH	7.8 ± 0.01^a^	7.9 ± 0.62^a^	8.02 ± 0.31a	8.02 ± 0.48^a^
Dissolved Oxygen (mg/l)	6.5 ± 0.81^a^	6.4 ± 0.33^a^	6.8 ± 0.32^a^	7.1 ± 0.23^a^
NH3 (mg/l)	0.01 ± 0.01	0.01 ± 0.01	0.01 ± 0.02	0.01 ± 0.01
NH4	0.25 ± 0.32^b^	0.38 ± 0.48^a^	0.39 ± 0.81^a^	0.41 ± 0.73^a^
NO_2_	0.042 ± 0.43^a^	0.037 ± 0.22^a^	0.040 ± 0.52^a^	0.046 ± 0.62^a^

Different small alphabetic within the same sampling time (rows) denote significant differences between means. T1= (control group), T2= (0.1 g/kg Ecobiol plus^®^), T3= (0.2 g/kg Ecobiol plus^®^), T4= (0.4 g/kg Ecobiol plus^®^)

### Growth parameters

At the eight-week mark, each mimic had 25 fish weighed using an electronic balance. The criteria were the (FCR), weight increase, and final biomass [31]. The growth performance parameters were assessed using standard formulas: Weight Gain (WG) was calculated as WG (%) = [(Final Weight (g) - Initial Weight (g)) / Initial Weight (g)] × 100; Specific Growth Rate (SGR) as SGR (%/day) = [(ln(Final Weight) - ln(Initial Weight)) / Experimental Duration (days)] × 100; and Feed Conversion Ratio (FCR) as FCR = Feed Intake (g) / Weight Gain (g). Individual weight gain was determined by subtracting each fish’s initial weight from its final weight. Total weight gain (TWG) was defined as the cumulative weight increase for the entire group, normalized to the total number of fish per group. These parameters assessed the overall growth response to the dietary treatments. The Survival Rate (SR) was computed as SR (%) = (Final Number of Fish / Initial Number of Fish) × 100. The final biomass for each group was calculated by multiplying the average weight of the surviving fish (in grams) by the total number of survivors at the end of the trial. The average final weight was derived by dividing the total group weight by the number of fish in that group. This method effectively captured the combined influence of growth and survival on the performance outcomes of each treatment group.

 [32].

### Blood parameters

After twenty-four hours of starvation, the treated fish were ready for the last sampling. To lower stress levels during sampling, the fish were sedated by immersion in water containing 40 mg/L of tricaine methanesulfonate (MS-222, SyncaineR, Syndel, Canada). For tissue collection, the experimental fish were euthanized by immersion in 250 mg/L of MS-222 for 10 min [33]. Three fish per replication allowed a random selection of nine fish from each group for weighing. Blood was collected from the caudal vein and divided into two portions. One portion was promptly used for hematological assessments and differential leukocyte counts, with the samples stored in EDTA-heparinized tubes. The process adhered to the protocol described by Urbinate and Carneiro [34]. Fish were randomly sampled from the same replicate groups for specific analyses to ensure representative results while minimizing sampling-related stress. For each replicate tank, 3–4 fish were randomly selected for hematological and biochemical analysis, while a separate set of fish was used for histopathological and immune parameter assessments. This approach ensured that no fish was subjected to multiple sampling procedures, which could influence the validity of subsequent measurements. All fish in a replicate group were exposed to identical conditions, and the data from these representative subsets were treated as reflective of the respective treatment groups. Statistical analyses accounted for replication to ensure robust and reliable comparisons. Hematological parameters were assessed using various methods: a hemocytometer for counting erythrocytes (RBCs) and leucocytes (WBCs), hemoglobin concentration (Hb g/dL) determination following established protocols, the micro-hematocrit method for estimating (PCV%), (MCHC), and (MCV) carried out by Beckman Coulter of Fullerton, California, using an automated hematology analyzer model LH 750 [35]. Differential leucocytic counts were conducted using the techniques outlined by [4]. Albumins, globulin, total proteins (ALT), and (AST) were performed as prescribed by [35].

### Determination of immune parameters

Phagocytic activity (PA) was revealed using the method following Kawahara et al. [36]., calculated as PA (%) = (Macrophages containing yeast / Total number of macrophages) × 100. The Phagocytic Index (PI) was measured as PI = (Number of cells phagocytized / Number of phagocytic cells), following the protocols of [37, 38]. Serum lysozyme (LYZ) activity was evaluated using a turbidimetric assay with lyophilized Micrococcus lysodeikticus cells (Sigma-Aldrich, St. Louis, MN, USA), as described by Ellis [39].

### A. hydrophila challenge

The experimental infection procedure was performed following the method of Li et al. [40],, involving an intraperitoneal injection of 0.2 ml of *Aeromonas hydrophila* culture at a concentration of 3 × 10⁷ CFU/ml. The bacterial concentration was determined using the drop plate method, as described by [41]. The *A. hydrophila* strain was cultured on Tryptic Soy Agar (TSA) plates at 28 °C for 24 h, then suspended in sterile saline and serially diluted from 10² to 10⁷ CFU/ml. The final suspension was prepared for injections, with each fish receiving 0.5 ml of the bacterial dilution intraperitoneally. Fish were observed for two weeks post-injection, and mortalities were recorded twice daily as per Ibrahim et al. [42], Dead fish were collected for post-mortem examination. The lethal dose causing 50% mortality (LD50) was calculated according to Reed and Muench [43]; the LD50, denoting the lethal dose that causes the death of 50% of the treated fish, was calculated.

### Gene expression

RNA was extracted according to the instructions using the TRIzol reagent from Life Technologies in Gaithersburg, MD, USA. Here at Applied Biosystems in Foster City, CA, USA, we use the MultiScribe RT enzyme kit to synthesize cDNA. Real-time PCR was conducted in triplicate using the Power SYBR Green PCR Master Mix (Applied Biosystems, CA, USA) on a 7500 Real-Time PCR System (Applied Biosystems, Foster City, CA, USA). The thermocycling conditions included initial denaturation at 95 °C for 10 min, followed by 40 cycles of denaturation at 95 °C for 15 s, and annealing/extension at 60 °C for 1 min. The relative expression of target genes was compared to the control group, using β-actin as the normalization reference. The 2^−∆∆Ct method [44] was applied to calculate relative gene expression levels, and primer sequences are listed in Table 3.

**Table 3:** Primers sequences of genes analyzed in real-time PCR

Target genes	Forward primer	Reverse primer	Accession No.
*β. Actin*	CAGCAAGCAGGAGTACGATGAG	TGTGTGGTGTGTGGTTGTTTTG	EU887951.1
*IGF-1*	GTTTGTCTGTGGAGAGCGAGG	GAAGCAGCACTCGTCCACG	XM_00344805
*GHR*	CAGACTTCTACGCTCAGGTC	CTGGATTCTGAGTTGCTGTC	AY973232.1
*GPX*	CGCCGAAGGTCTCGTTATTT	TCCCTGGACGGACATACTT	NM_001279711.1
*SOD*	CCCTACGTCAGTGCAGAGAT	GTCACGTCTCCCTTTGCAAG	JF801727.1
*TNF-α*	GGAAGCAGCTCCACTCTGATGA	CACAGCGTGTCTCCTTCGTTCA	JF957373.1
*IL-1B*	CAAGGATGACGACAAGCCAACC	AGCGGACAGACATGAGAGTGC	XM_003460625.2

### Histopathological analysis

For histopathological analysis, samples from the intestine, hepatopancreas, kidney, and spleen were collected after anesthetizing fish with 40% ethyl alcohol. Five fish were randomly selected per treatment group (0.1, 0.2, and 0.4 g/kg of Ecobiol plus^®^) before and after the *A. hydrophila* challenge. Tissues were preserved in 10% formaldehyde for 24 h, dehydrated using a graded ethanol series (70% to absolute), cleared with xylene, and embedded in paraffin. Sections measuring 4–5 μm were stained with hematoxylin and eosin [4]. Dimensions of intestinal villi (length, width, crypt depth, and surface area) were quantified using ImageJ software (v. 1.53k) (Bethesda, MD, USA). Data from 10 randomly selected villi and crypts in 5 cross-sections were analyzed with one-way ANOVA in SPSS v.22, with significance set at *p* < 0.05. Results are expressed as means ± standard error.

### Statistical analysis

The Shapiro-Wilk test was used to assess data normality, while Levene’s test was employed to verify homogeneity of variance. Statistical comparisons among experimental groups were conducted using one-way ANOVA (SPSS version 22, SPSS Inc., IL, USA). Duncan’s post-hoc test was applied for further analysis when significant differences were identified. A p-value of less than 0.05 was deemed statistically significant. Results are presented as means ± standard error.

## Results

### Growth and blood parameters

The impacts of Ecobiol plus^®^ on the growth performance of *O. niloticus* were evaluated. All treated groups showed significant improvements in final weight, weight gain, and total weight gain compared to the control group (T1), with the T4 group exhibiting the highest values (*p* < 0.05; Table 4). Additionally, the feed conversion ratio (FCR) significantly improved across all treated groups, with T4 showing the most favorable FCR (*p* < 0.05; Table 5). However, feed intake remained consistent across all groups, with no significant differences observed (*p* > 0.05; Table 5). No substantial differences were detected between the treated and control groups in RBCs, PCV, and blood indices. However, (Hb) levels were substantially elevated in the T4 group compared to the other groups (*p* < 0.05; Table 6). For differential leukocyte counts, WBCs, lymphocytes, and monocytes were substantially elevated (*p* < 0.05) in all treated groups, with the highest values observed in the T3 and T4 groups. In contrast, basophil, eosinophil, and heterophil counts did not differ substantially among the groups (*p* > 0.05; Table 7). Biochemical analysis showed significantly higher globulin and total protein levels in all treated groups than the control, with the T4 group exhibiting the highest values (*p* < 0.05; Table 8). In contrast, albumin, ALT, and AST levels remained consistent across all groups, with no significant differences observed (*p* > 0.05; Table 8).

**Table 4:** The effect of Ecobiol plus^®^ on growth performance of *Oreochromis niloticus*

Variables		Groups				
	T1	T2	T3	T4		
Initial Weight Fish	37.5 ± 1.8 ^a^	35 ± 1 ^a^	35.67 ± 1.53 ^a^	33.5 ± 1.8 ^a^		
Fish Final Weight	75.26 ± 3.87^c^	75.46 ± 1.77 ^b^	76.04 ± 3.24 ^a^	77.96 ± 5.53 ^a^		
SGR (%/day)	1.24^c^	1.37^b^	1.35^b^	1.53^a^		
Fish Weight Gain	35.76 ± 2.08 ^c^	38.5 ± 2.3 ^b^	37.47 ± 1.82 ^b^	42.46 ± 3.18 ^a^		
Total Weight Gain	551.93 ± 50.88 ^b^	864.62 ± 75.93 ^a^	863.53 ± 42.26 ^a^	909.11 ± 76.01 ^a^		
Feed intake	56.85 ± 1.85 ^a^	55.67 ± 2.08 ^a^	56.13 ± 2.99 ^a^	59.93 ± 4.01 ^a^		
FCR	1.59 ± 0.04 ^a^	1.45 ± 0.05 ^bc^	1.5 ± 0.01 ^b^	1.41 ± 0.03 ^c^		
Initial Biomass	937.5 ± 45.07 ^a^	875 ± 25 ^a^	837.67 ± 38.19 ^a^	891.5 ± 45.07 ^a^		
Final Biomass	1530.09 ± 81.78 ^b^	1785.85 ± 55.73 ^a^	1825.04 ± 77.65 ^a^	1793.61 ± 154.89 ^a^		
Initial number	25 ± 0.00	25 ± 0.00	25 ± 0.00	25 ± 0.00		
Final number	20.33 ± 0.58 ^b^	23.67 ± 0.58 ^a^	23 ± 0 ^a^	24 ± 1 ^a^		
Survival rate %	81.32	94.86	92	96		

Different small alphabetic within the same sampling time (rows) denote significant differences between means. T1= (control group), T2= (0.1 g/kg Ecobiol plus^®^), T3= (0.2 g/kg Ecobiol plus^®^), T4= (0.4 g/kg Ecobiol plus^®^), FCR = Feed conversion ratio

**Table 5:** Effect of Ecobiol plus^®^ on haematological parameters of *Oreochromis Niloticu*

Variables	Groups			
	T1	T2	T3	T4
RBCS	2.4 ± 0.01 ^a^	2.33 ± 0.02 ^a^	2.42 ± 0.04 ^a^	2.49 ± 0.11 ^a^
HB	7.25 ± 0.01 ^bc^	7.07 ± 0.03 ^c^	7.43 ± 0.04 ^ab^	7.66 ± 0.21 ^a^
MCV	96.03 ± 0.28 ^a^	98.93 ± 0.91 ^a^	97.1 ± 1.22 ^a^	98.63 ± 1.36 ^a^
PCV	23 ± 0 ^a^	23 ± 0 ^a^	23.5 ± 0.71 ^a^	24.5 ± 0.71 ^a^
MCH	30.25 ± 0.06 ^a^	30.41 ± 0.4 ^a^	30.71 ± 0.71 ^a^	30.84 ± 0.46 ^a^
MCHC	31.5 ± 0.03 ^a^	30.74 ± 0.13 ^a^	31.63 ± 1.13 ^a^	31.27 ± 0.04 ^a^

Different small alphabetic within the same sampling time (rows) denote significant differences between means. T1= (control group), T2= (0.1 g/kg Ecobiol plus^®^), T3= (0.2 g/kg Ecobiol plus^®^), T4= (0.4 g/kg Ecobiol plus^®^), RBCs = Red Blood Cells, HB = Haemoglobin, PCV = Packed Cell Volume, MCV = Mean corpuscular volume, MCH = Mean corpuscular hemoglobin, MCHC = Mean corpuscular hemoglobin concentration

**Table 6:** Effect of Ecobiol plus^®^ on the differential leukocytic count of *Oreochromis niloticus*

Variables	Groups			
	T1	T2	T3	T4
WBCs	24.49 ± 0.5 ^b^	30.17 ± 0.02 ^a^	29.06 ± 1.55 ^a^	28.05 ± 0.1 ^a^
Basophil	0.15 ± 0.21 ^a^	0.13 ± 0.18 ^a^	0 ± 0 ^a^	0.28 ± 0 ^a^
Eosinophil	0.15 ± 0.21 ^a^	0.37 ± 0.18 ^a^	0.15 ± 0.21 ^a^	0.28 ± 0 ^a^
Monocyte	1.84 ± 0.21 ^c^	2.11 ± 0.01 ^b^	2.62 ± 0.13 ^a^	2.72 ± 0.21 ^a^
Lymphocyte	19.59 ± 0.08 ^c^	22.16 ± 0.48 ^b^	23.68 ± 1.05 ^ab^	24.29 ± 0.23 ^a^
Heterophil	2.87 ± 0.21 ^a^	2.57 ± 0.11 ^a^	2.62 ± 0.13 ^a^	3.23 ± 0.59 ^a^

Different small alphabetic within the same sampling time (rows) denote significant differences between means. T1= (control group), T2= (0.1 g/kg Ecobiol plus^®^), T3= (0.2 g/kg Ecobiol plus^®^), T4= (0.4 g/kg Ecobiol plus^®^), WBCs = White Blood Cells

**Table 7:** Effect of Ecobiol plus^®^ on biochemical analysis of *Oreochromis niloticus*

Variables	Groups			
	T1	T2	T3	T4
Globulin	2 ± 0.05 ^c^	2.17 ± 0.05 ^ab^	2.06 ± 0.06 ^bc^	2.23 ± 0.03 ^a^
Albumin	1.32 ± 0.02 ^a^	1.33 ± 0.02 ^a^	1.33 ± 0.04 ^a^	1.33 ± 0.02 ^a^
TP	3.32 ± 0.03 ^c^	3.48 ± 0.03 ^ab^	3.39 ± 0.04 ^bc^	3.56 ± 0.06 ^a^
AST	28.6 ± 0.4 ^a^	28.9 ± 0.05 ^a^	28.02 ± 0.05 ^a^	28.3 ± 0.04 ^a^
ALT	29 ± 0.5 ^a^	30.59 ± 0.71 ^a^	28.73 ± 0.59 ^a^	29.6 ± 0.05 ^a^

Different small alphabetic within the same sampling time (rows) denote significant differences between means. T1= (control group), T2= (0.1 g/kg Ecobiol plus^®^), T3= (0.2 g/kg Ecobiol plus^®^), T4= (0.4 g/kg Ecobiol plus^®^), ALT = Alanine aminotransferase, AST = Aspartate aminotransferase

**Table 8:** Effect of Ecobiol plus^®^ on immunity of *Oreochromis niloticus*

Variables	Groups			
	T1	T2	T3	T4
lysozyme	10.09 ± 0.05 ^b^	11.28 ± 0.04 ^a^	11.1 ± 0.08 ^a^	11.09 ± 0.1 ^a^
PI	1.2 ± 0.02 ^b^	1.27 ± 0.09 ^a^	1.32 ± 0.04 ^a^	1.3 ± 0.02 ^a^
PA	10.18 ± 0.04 ^c^	10.05 ± 0.04 ^c^	10.51 ± 0.03 ^b^	11.07 ± 0.08 ^a^

Different small alphabetic within the same sampling time (rows) denote significant differences between means. T1= (control group), T2= (0.2 g/kg Ecobiol plus^®^), T3= (0.4 g/kg Ecobiol plus^®^), T4= (0.8 g/kg Ecobiol plus^®^)

### Immunological parameters

Lysozyme activity and phagocytic index (PI) were substantially superior in all treated groups associated to the control (*p* < 0.05). (PA) was increased dramatically in the T3 and T4 treated one associated to T2 and T1 (*p* < 0.05; Table 9). The LD50 of *Aeromonas hydrophila* was determined to be 3 × 10⁷ CFU/mL. This value represents the bacterial concentration that resulted in 50% mortality in the untreated control group. The determination of LD50 acts as a scale for assessing the protective effects of Ecobiol plus^®^ against *A. hydrophila* infection. Fish groups supplemented with Ecobiol plus^®^ exhibited significantly improved survival rates, even at the LD50 challenge dose. Specifically, the 0.4 g/kg group achieved a survival rate of 96%, highlighting the efficacy of the probiotic in mitigating the pathogenic effects of *A. hydrophila* (Suppl Table 1).

**Table 9:** Effect of Ecobiol plus^®^ on immunity of *Oreochromis niloticus* challenged with *A. Hydrophila*

Variables	Groups			
	T1	T2	T3	T4
lysozyme	10.18 ± 0.08 ^b^	11.14 ± 0.23 ^a^	11.18 ± 0.06 ^a^	11.04 ± 0.08 ^a^
PI	1.33 ± 0.04 ^a^	1.29 ± 0.01 ^a^	1.33 ± 0.05 ^a^	1.27 ± 0.05 ^a^
PA	10.91 ± 0.96 ^a^	10.17 ± 0.12 ^a^	11.16 ± 0.14 ^a^	10.55 ± 0.13 ^a^

Different small alphabetic within the same sampling time (rows) denote significant differences between means. T1= (control group), T2= (0.1 g/kg Ecobiol plus^®^), T3= (0.2 g/kg Ecobiol plus^®^), T4= (0.4 g/kg Ecobiol plus^®^)

Post-challenge with *A. hydrophila*, lysozyme activity was markedly improved (*p* < 0.05) in all groups treated with Ecobiol plus^®^ associated with the control. However, no significant phagocytic index or activity differences were observed across the groups (*p* > 0.05; Table 10).

**Table 10:** Morphometric analysis of the intestine of *O. Niloticus* treated fed on diets containing different concentrations of Ecobiol plus^®^

Groups	T1	T2	T3	T4	*P*. value
Parameters					
Villi length (µm)	321.3 ± 12.32^b^	345.3 ± 15.06^b^	629.4 ± 38.15^a^	714.3 ± 32.53^a^	< 0.0001
Crypt depth (µm)	70.58 ± 3.41^bc^	55.38 ± 4.04^c^	80.39 ± 6.84^ab^	97.44 ± 7.36^a^	< 0.0001
villi width (µm)	87.84 ± 5.97^c^	113.0 ± 7.18^bc^	131.21 ± 5.03^ab^	144.8 ± 8.65^a^	< 0.0001
Villi length/crypt depth	4.92 ± 0.61^c^	7.01 ± 0.81^bc^	8.69 ± 0.56^ab^	9.89 ± 0.74^a^	< 0.0001
Villi surface area (µm^2^)	273142 ± 198^b^	307721 ± 232^b^	656245 ± 314^a^	891690 ± 418^a^	< 0.0001

The presented data represent the mean ± SE. The values with different superscripts in the same row indicate significant differences (*p* < 0.05). T1= (control group), T2= (0.1 g/kg Ecobiol plus^®^), T3= (0.2 g/kg Ecobiol plus^®^), T4= (0.4 g/kg Ecobiol plus^®^)

### Gene expression

**Fig. 1:**
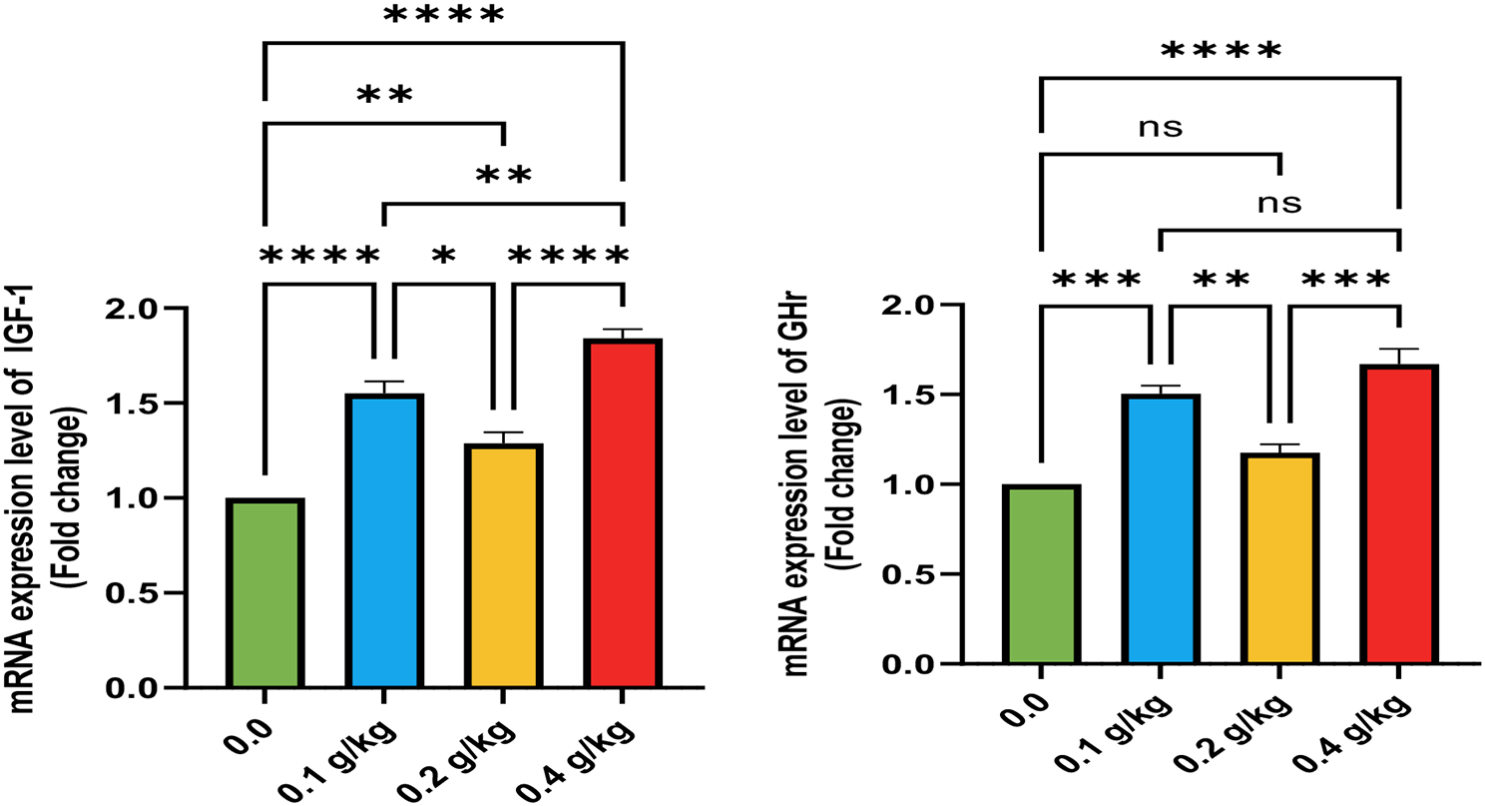
Gene expression of the liver (IGF-1) and (GHR) of Nile tilapia in different groups supplemented with different doses of Ecobiol plus^®^ for 8 weeks. * denote notable variation between groups (*P* < 0.05), ns = denote no notable variation between groups

**Fig. 2:**
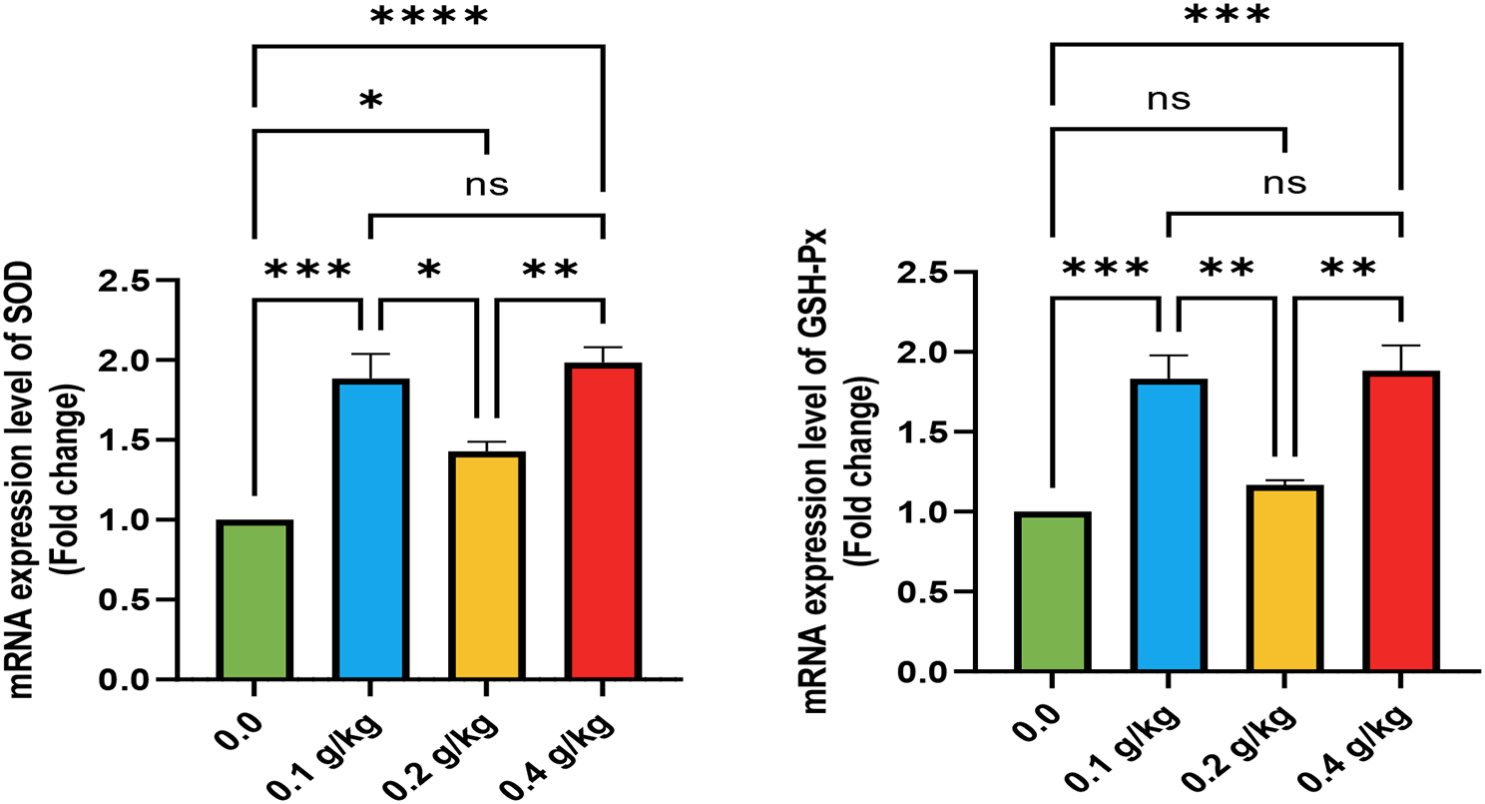
Gene expression of liver (SOD) and (GSH-PX) of Nile tilapia in different groups supplemented with different doses of Ecobiol plus ® for 8 weeks. * denote notable variations between groups (*P* < 0.05), ns = denote no notable variations between groups

**Fig. 3:**
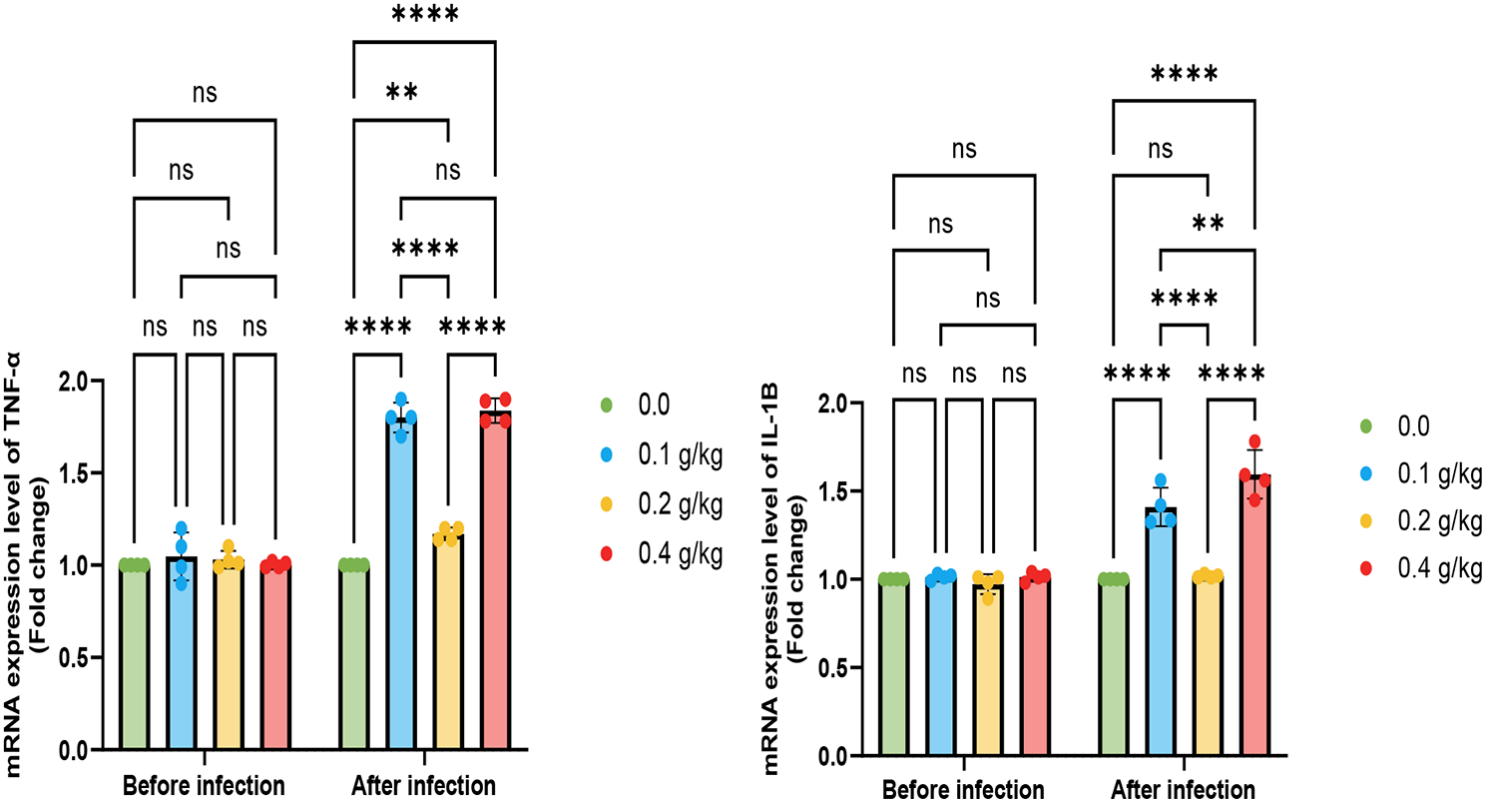
Gene expression of liver (TNF-α) and (IL-1β) of Nile tilapia pre and post challenge with *A. hydrophila* in different groups supplemented with different Ecobiol plus ® for 8 weeks. * denote notable variations between groups (*P* < 0.05), ns = denote no notable variations between groups

The mRNA expression levels of *IGF-1*, *GSH-Px*, and *GHR* were significantly elevated in the liver of fish from the T2 and T4 groups, supplemented with 0.1 and 0.4 g/kg of Ecobiol plus^®^, respectively (*p* < 0.05; Figs. 1 and 2). Additionally, all Ecobiol plus^®^-treated groups displayed markedly higher *TNF-α* mRNA expression (*p* < 0.05), with no significant differences before the *A. hydrophila* challenge. Following the challenge, *IL-1β* expression was significantly upregulated in the T2 and T4 groups, with the T4 group showing the highest levels (*p* < 0.05; Fig. 3). The upregulation of genes (*IGF-1*, *GSH-Px*, *GHR*, *SOD*, *TNF-α*, and *IL-1β*) in the T2 and T4 groups followed a consistent pattern, though expression magnitudes varied. Notably, *IGF-1* and *GHR* exhibited more substantial increases compared to *SOD* and *GSH-Px*, reflecting the interplay between growth, antioxidant, and immune pathways activated by probiotic supplementation.

### Histopathological result

**Fig. 4:**
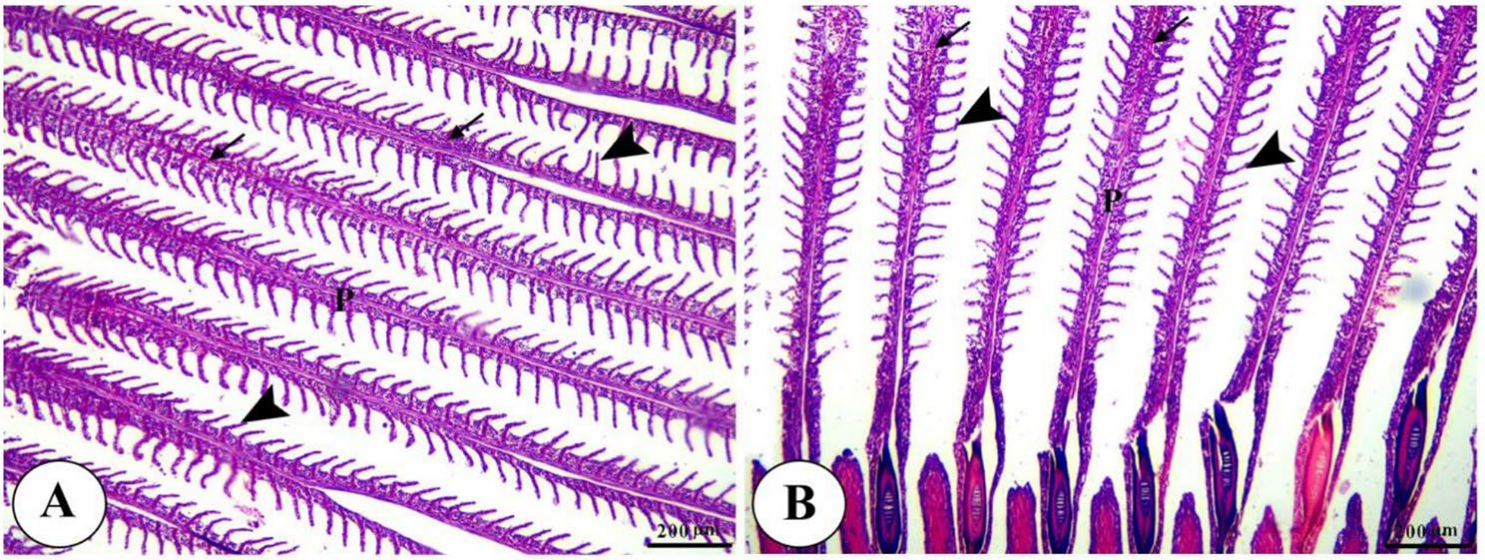
Photomicrograph of H&E-stained panel of gills of control (**A**) and Ecobiol plus^®^ (**B**) treated groups showing intact primary gill lamellae (P), secondary lamellae (arrow heads), average size and intact branchial blood vessels

**Fig. 5:**
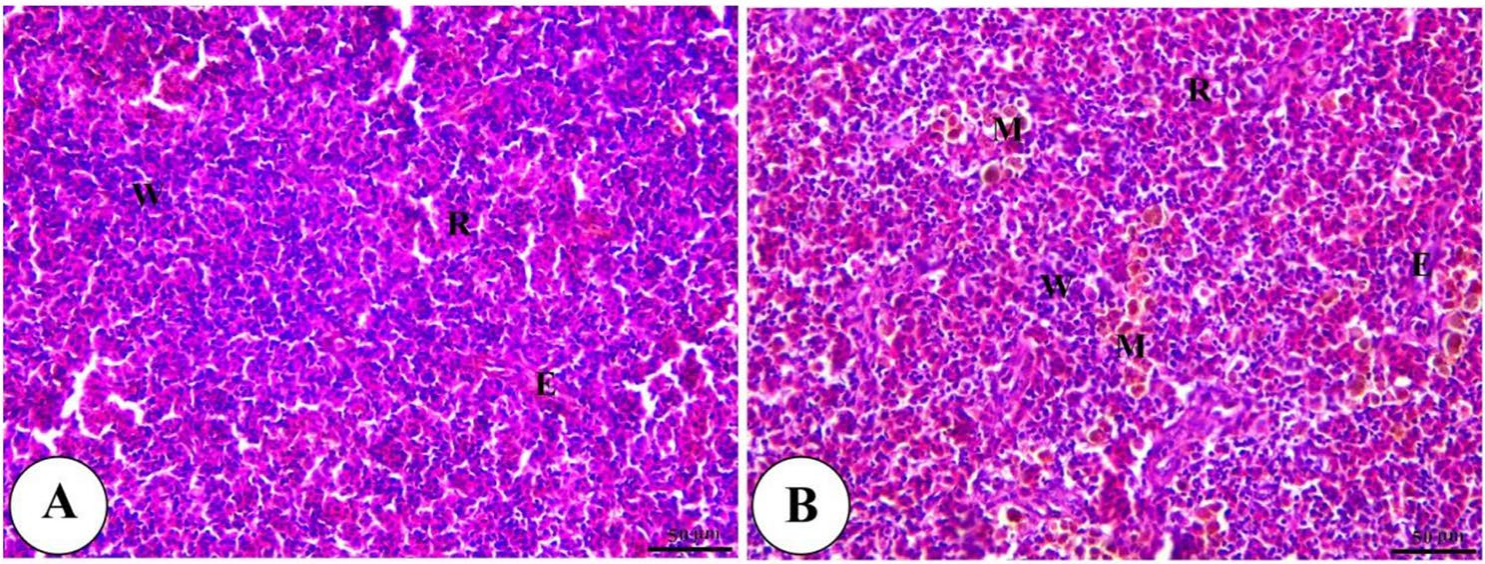
Photomicrograph of H&E stained panel of spleen of control (**A**) and Ecobiol plus^®^ (**B**) treated groups showing normal splenic architecture with interconnected red (R) and white (W) pulp in addition to ellipsoid (E) and melanomacrophage (M)

**Fig. 6:**
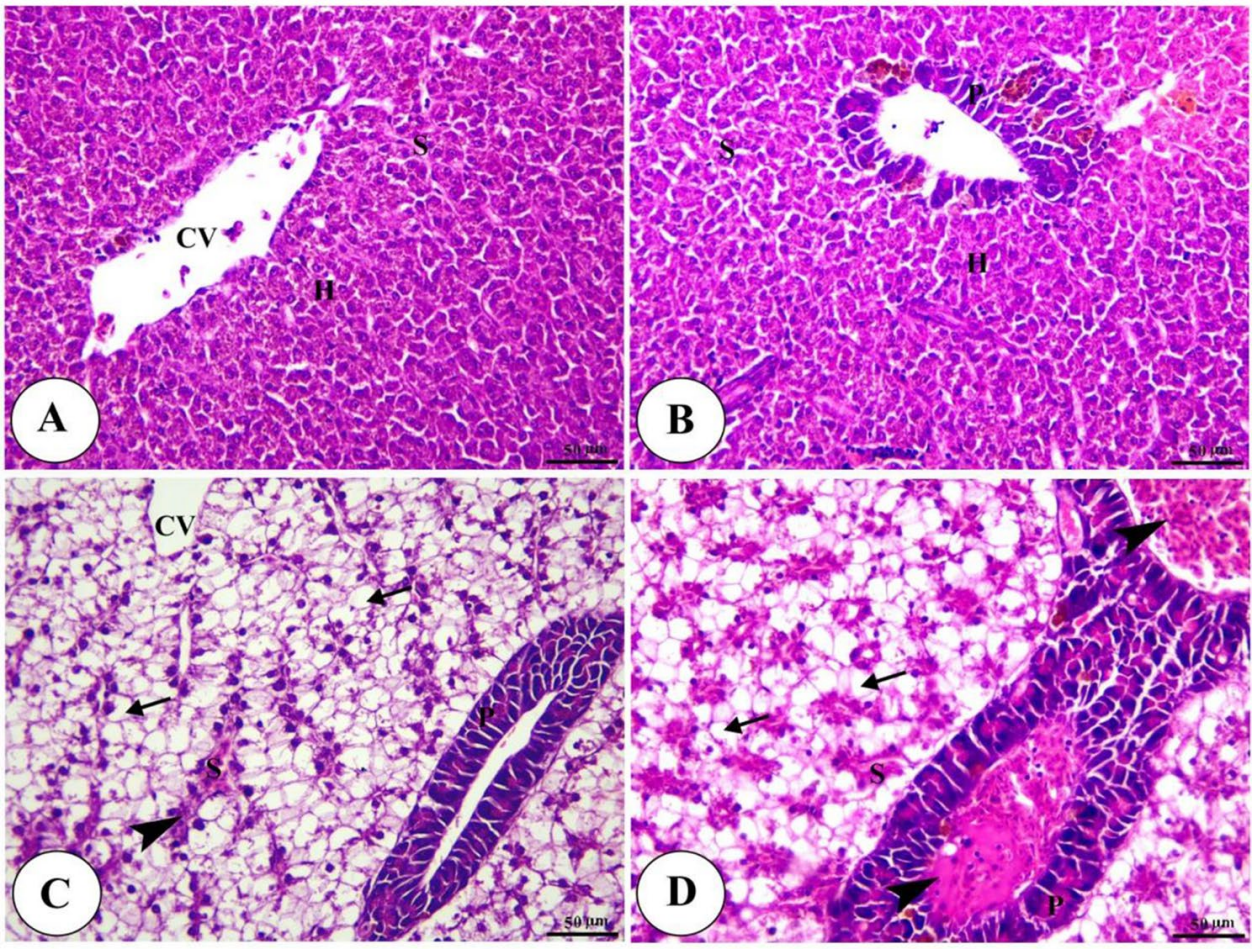
Photomicrograph of H&E stained panel of hepatopancreas of control (**A**), Low (**B**), medium (**C**) and high (**D**) Ecobiol plus^®^ treated groups showing normal hepatic architecture in which hepatocytes (**H**) with centrally located nuclei are arranged in cord-like pattern and separated by blood sinusoids (S) radiating from central vein (CV) in addition to presence of pancreatic acini (P). Micro and microsteatosis of hepatocytes (arrows) and congestion of blood vessels (arrowheads) were evident, especially in Ecobiol plus^®^ treated groups

**Fig. 7:**
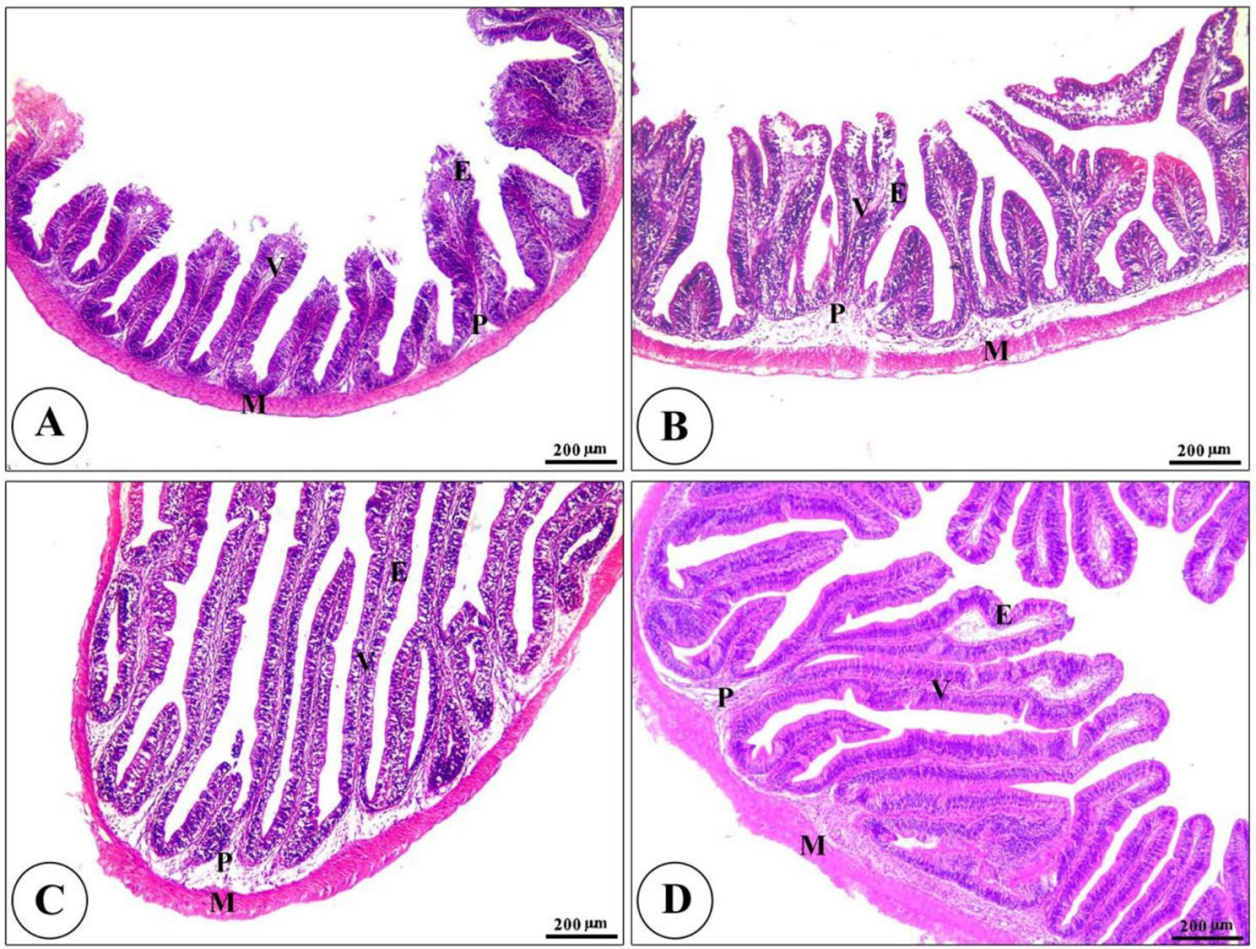
Photomicrograph of H&E stained panel of the anterior part of the intestine of control (**A**), Low (**B**), medium (**C**), and high (**D**) Ecobiol plus^®^ treated groups showing intact intestinal villi (V), which were elongated and show some branching, especially with a high concentration of Ecobiol plus^®^. The villi were lined by simple columnar epithelium with goblet cells (**E**) tested on lamina propria of loose C.T (P) and lamina muscularis (M), which were covered externally by tunica serosa

**Fig. 8:**
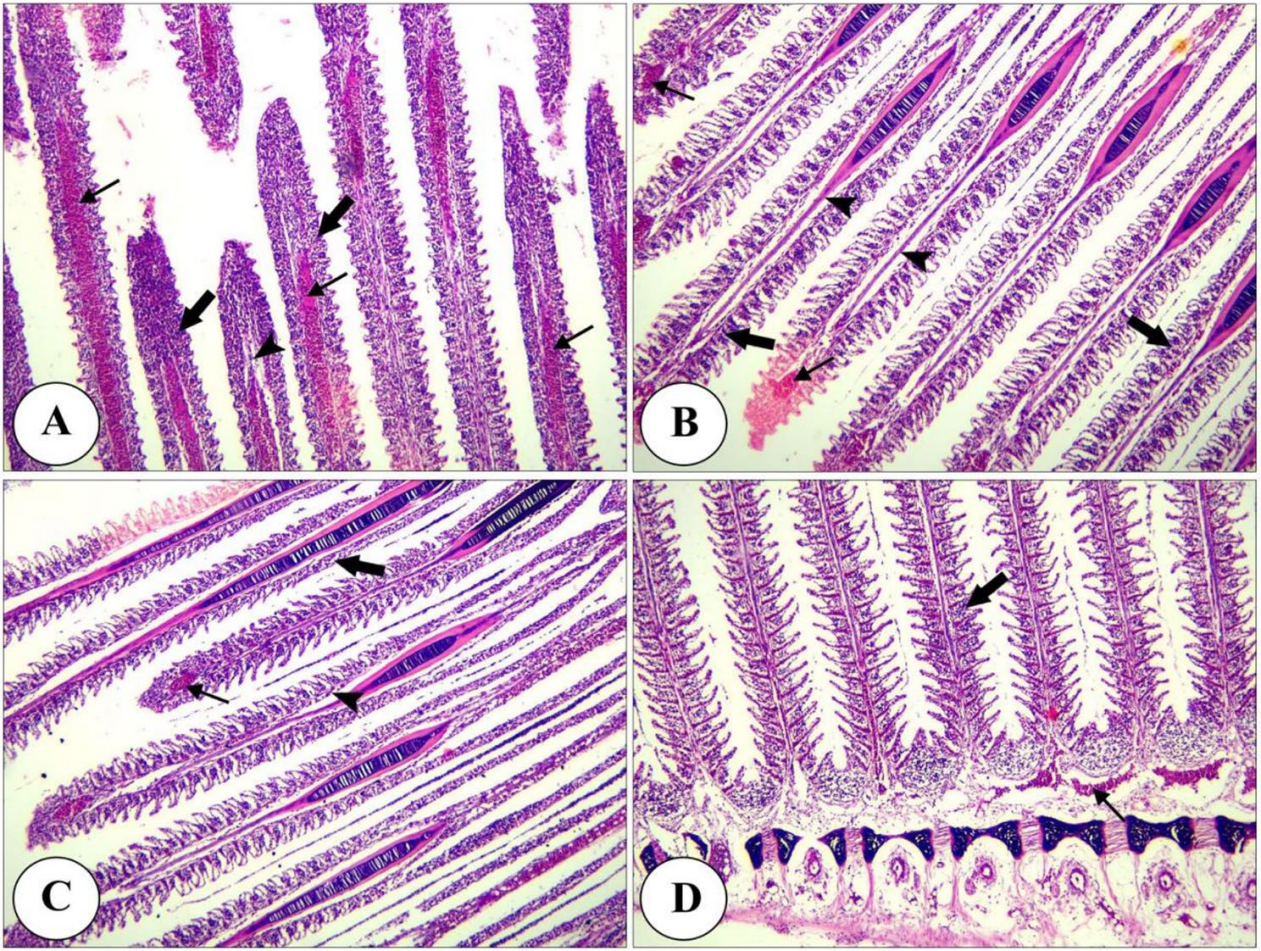
Photomicrograph of H&E stained panel of gills of post-challenged control (**A**), low (**B**), medium (**C**), and High (**D**) Ecobiol plus^®^ treated groups showing hyperplasia and fusion of secondary lamellae (thick arrows), congestion of branchial blood vessels (thin arrows), edema in primary and secondary lamellae (arrowheads)

**Fig. 9:**
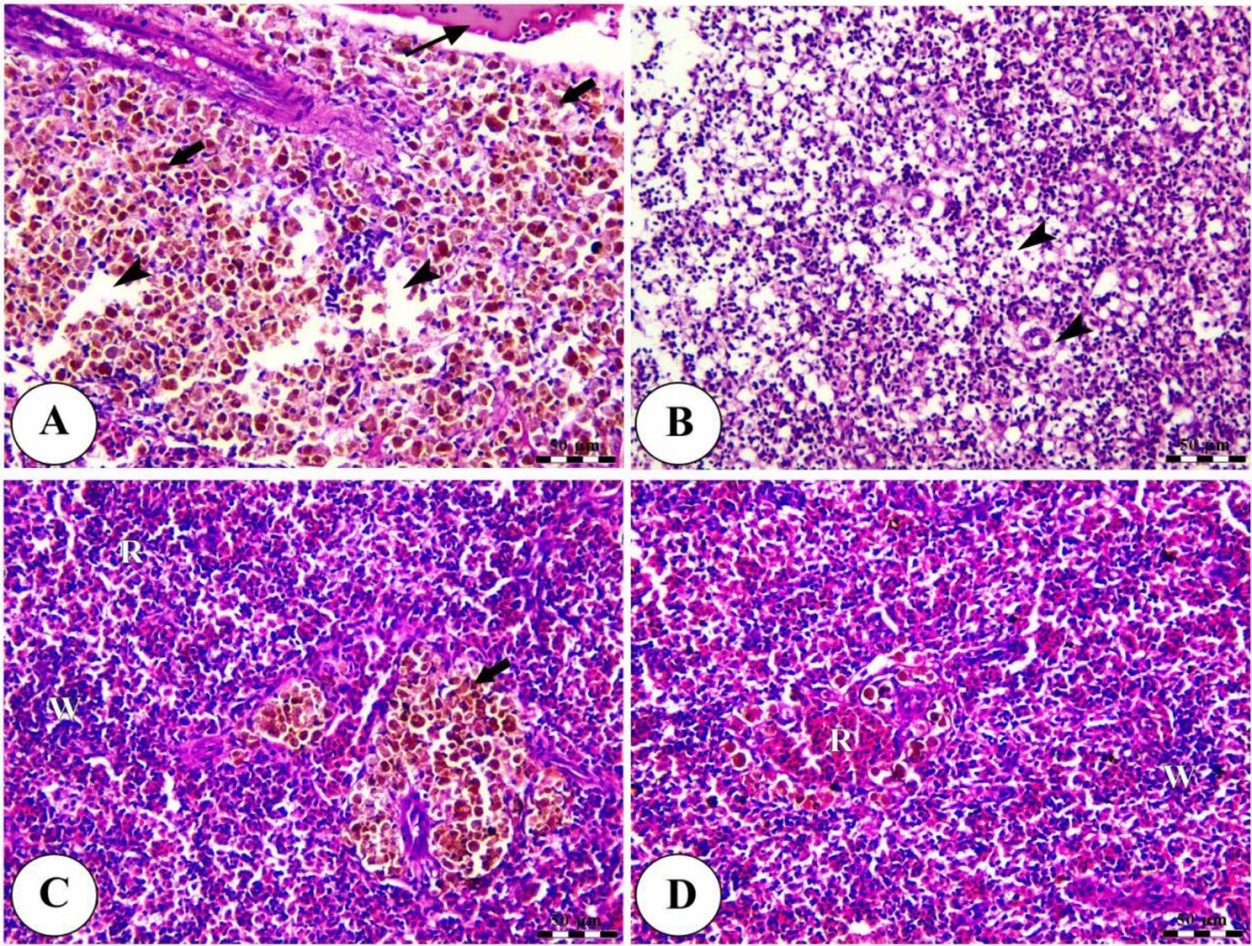
Photomicrograph of H&E stained panel of spleen of post-challenged control (**A**), low (**B**), medium (**C**), and High (**D**) Ecobiol plus^®^ treated groups showing increase in melanomacrophage centers in control, medium Ecobiol plus^®^ groups (thick arrows), congestion and hemolysis of splenic blood vessels (thin arrows) in addition to edema, depletion of white pulp and necrosis of splenic parenchyma in control and low Ecobiol plus^®^ groups (arrowheads) while the medium and high groups appeared intact with mixed red (R) and white (W) pulp

**Fig. 10:**
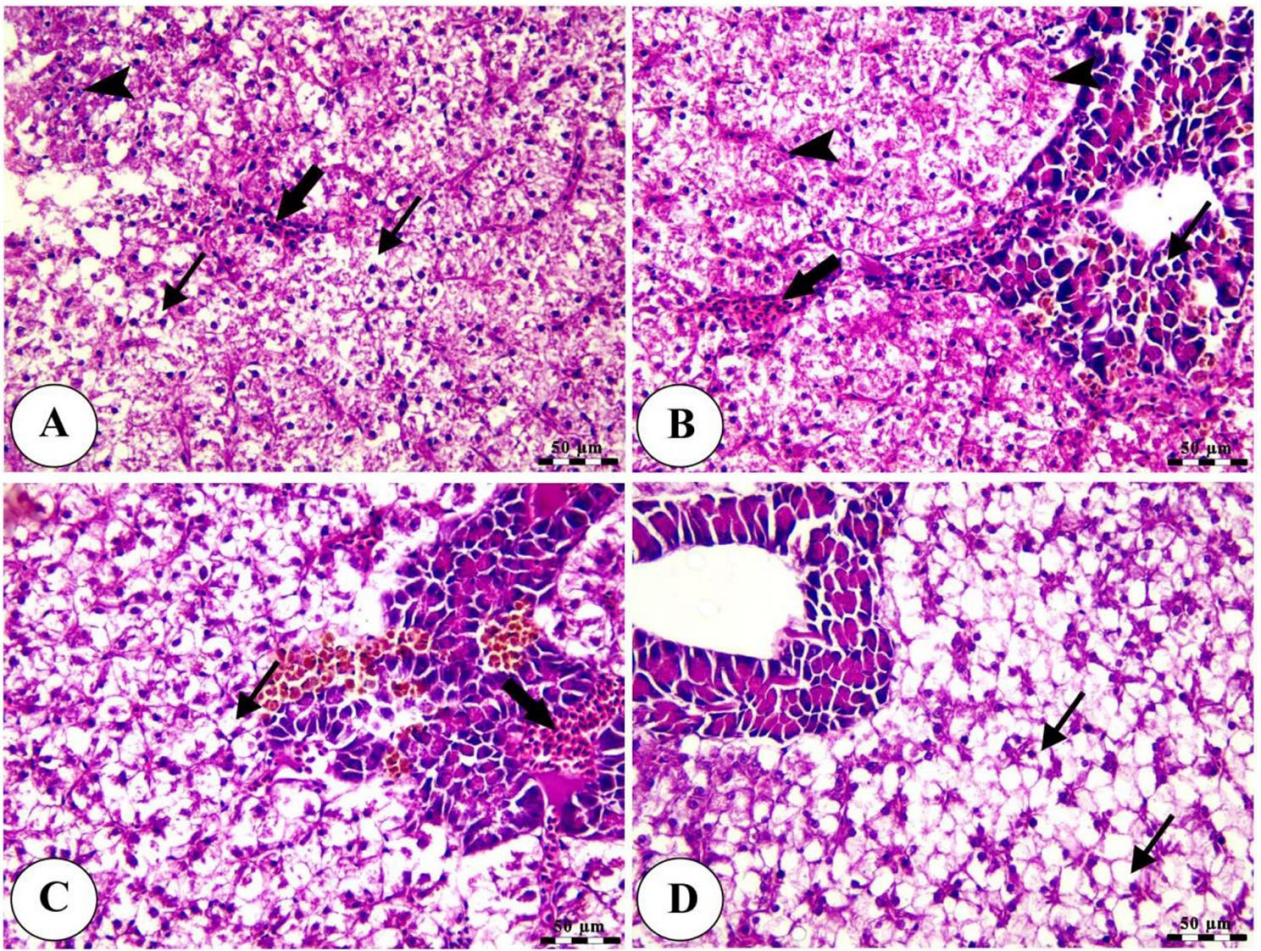
Photomicrograph of H&E stained panel of the hepatopancreas of post-challenged control (**A**), low (**B**), medium (**C**), and High (**D**) Ecobiol plus^®^ treated groups showing hepatocellular necrosis (arrowheads), congestion of hepatic and pancreatic blood vessels (thick arrows) in addition to vacuolar degeneration of hepatic and pancreatic cells (thin arrows)

**Fig. 11:**
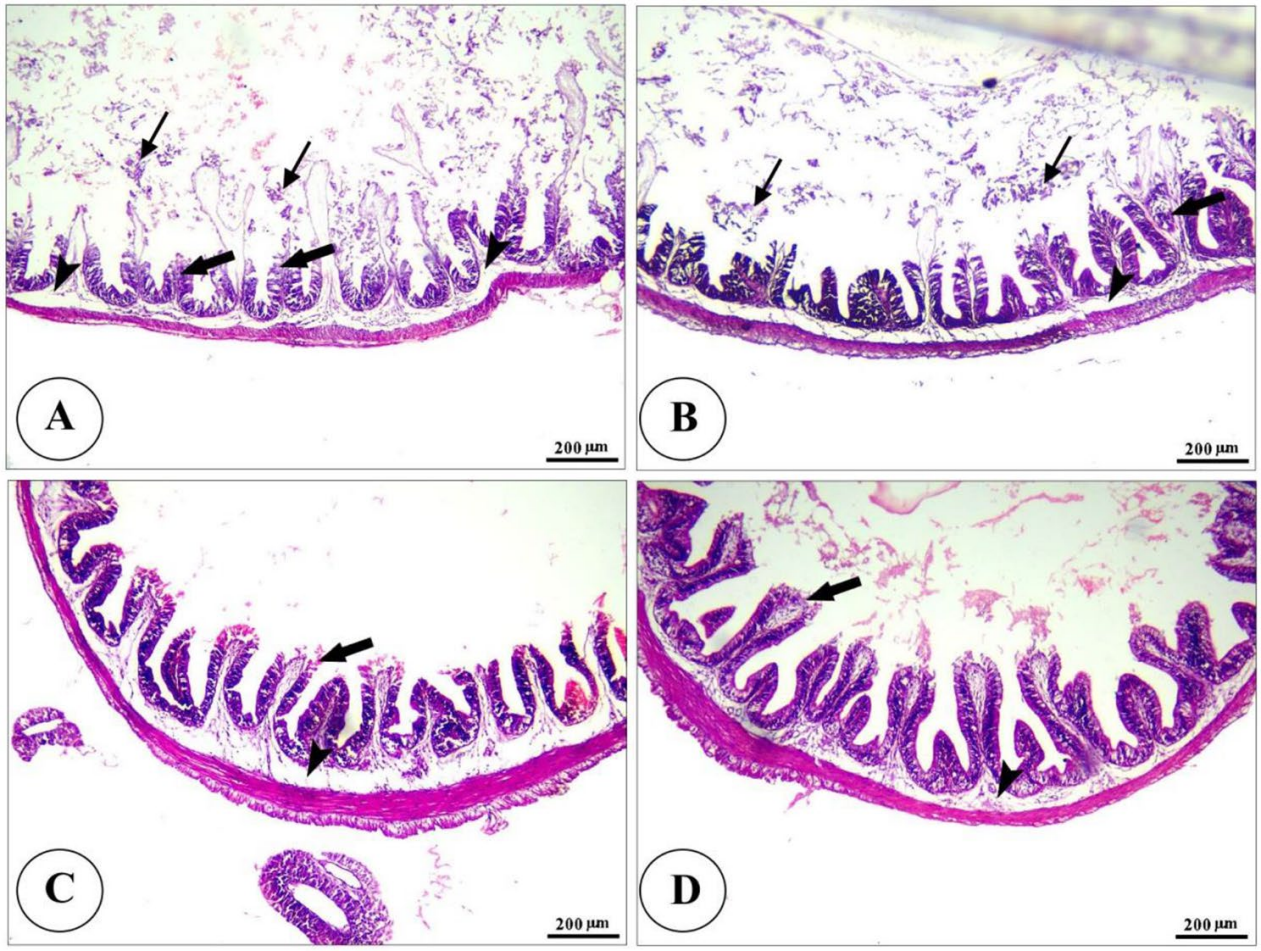
Photomicrograph of H&E stained panel of the anterior part of the intestine of post-challenged control (**A**), low (**B**), medium (**C**), and High (**D**) Ecobiol plus^®^ treated groups showing sloughing of the apical part of intestinal villi (thin arrows), degeneration of lamina epithelial (thick arrows) and edema in the lamina propria (arrowheads)

Histopathological analysis revealed intact gills (Fig. 4) and spleen (Fig. 5) in both control and Ecobiol plus^®^-treated groups. The hepatopancreas showed normal structure in control and low-dose groups. At the same time, medium and high doses caused mild to moderate vacuolation and vascular congestion in a dose-dependent manner (Fig. 6). Intestinal examination showed improved villi structure and morphometric parameters, particularly at higher Ecobiol plus^®^ concentrations (Fig. 7; Table 10). In challenged, untreated controls, gills exhibited severe damage, which was progressively alleviated with Ecobiol plus^®^, appearing intact at high doses (Fig. 8). The gills, spleen, hepatopancreas, and anterior intestine showed notable differences between the control and treated groups. Gills: The gills of the control non-treated group showed severe hyperplasia, fusion of secondary lamellae, and congestion. In a dose-dependent way, these pathogenic results were diminished., with the gills of the T4 group appearing intact (Fig. 8). Spleen The control and low-concentration groups exhibited severe congestion, hemolysis, and necrosis, while the medium and high-concentration groups showed reduced pathological changes. The T4 group showed normal splenic structure (Fig. 9). Hepatopancreas: The control group exhibited necrosis, mononuclear cell infiltration, and vacuolar degeneration of hepatic and pancreatic tissue. These lesions were reduced in the T3 and T4 groups, which showed only mild vacuolar degeneration (Fig. 10). Intestine: The control and low-concentration groups displayed degenerative alterations in the intestinal epithelium and sloughing of villi. In contrast, the medium and high-concentration groups (T3 and T4) showed marked improvement, with elongated and branched villi (Fig. 11). According to the results shown in Table 10, the villi’s breadth, length, crypt depth, a ratio of villi length to crypt depth, and surface area all improved substantially as the dosage increased.

## Discussion

Probiotics and prebiotics in feed can boost aquaculture output, reduce mortality, and mitigate biotic and abiotic stressors [45]. In this study, the dietary supplementation of *Bacillus amyloliquefaciens* (Ecobiol plus^®^) substantially enhanced growth performance, immune responses, gene expression, and intestinal morphology in Nile tilapia. These findings underline the potential of functional feed additives as sustainable alternatives to antibiotics in aquaculture, addressing both productivity and environmental concerns [46]. The study revealed that all growth parameters were significantly enhanced in Ecobiol plus^®^-treated groups, with the highest increases noticed in the T4 group (0.4 g/kg Ecobiol plus^®^). These enhancements are attributed to better feed utilization, reduced feed conversion rates, and improved digestibility of diet components, leading to reduced production costs [47, 48]. The ability of probiotics to modulate gut microbiota diversity enhances nutrient absorption and growth performance, consistent with previous findings [23, 49]. Specific probiotic strains, including *B. amyloliquefaciens*, may act as supplementary nutritional sources, synthesizing essential nutrients and stimulating appetite through feed purification and vitamin synthesis [49]. Similar trends have been reported in Nile tilapia and *Litopenaeus vannamei* post-larvae, where enhanced intestinal microbiota improved nutrient metabolism and immunity [50]. These findings reinforce the role of gut microbiota in mediating nutrient assimilation and systemic immune responses [51]. Hematological analysis indicated no significant variation in RBC counts among the treated and control groups, consistent with ElSabagh et al. [47]. However, the T4 group exhibited a substantial increase in hemoglobin (Hb) levels, likely reflecting higher oxygen transport to meet the energy demands of improved growth [52]. Enhanced WBC and lymphocyte counts were observed in all Ecobiol plus^®^-treated groups, indicative of an elevated immune response triggered by the probiotic supplementation [53]. The observed erythrogram improvements may be linked to probiotics’ hepatoprotective and hepatostimulatory properties [54], as well as enhanced iron absorption facilitated by organic acid secretion in the gastrointestinal tract [55]. The biochemical parameters demonstrated hyperproteinemia and hyperglobulinemia in T4-treated fish, suggesting an enhanced innate immune response [56]. Globulin, a precursor of antibodies, is a biomarker for improved immunity, corroborating prior findings in the Nile tilapia-fed probiotic diets [57, 58]. Variations in ALT and AST levels were minimal, indicating the hepatoprotective effects of Ecobiol plus^®^, as lower enzyme leakage points to intact liver cell membranes [59].

Ecobiol plus^®^ significantly amplified immunological responses, with the highest lysozyme activity and phagocytic indices detected in the T4 group. Lysozyme, a critical component of innate immunity, protects fish against infections through its bactericidal action [4, 60]. Probiotic administration likely stimulated lysozyme-secreting cells, enhancing non-specific immunity [61]. These findings are consistent with prior studies in Nile tilapia, rainbow trout, and *L. vannamei*, demonstrating the broad-spectrum efficacy of Bacillus probiotics in aquaculture [62].The enhanced immune responses observed in Ecobiol plus^®^-treated groups were maintained after the *(A) hydrophila* challenge. Lysozyme and phagocytic activity were significantly increased, consistent with prior research linking *(B) amyloliquefaciens* supplementation to improved bactericidal and lysozyme activity [63]. These effects likely contributed to reduced mortality and enhanced disease resistance in treated fish, emphasizing the role of feed additives in improving resilience under pathogenic stress. The LD50 of *Aeromonas hydrophila* was determined to be three × 10⁷ CFU/mL, consistent with reported virulence levels in aquaculture species [40]. Fish supplemented with Ecobiol plus^®^ showed significantly higher survival rates at the LD50 dose, with the 0.4 g/kg group achieving 96% survival contrasted to 81.32% in the control. This highlights the potential of Ecobiol plus^®^ as a prophylactic additive to increase immune response and disease resistance in aquaculture systems.

Probiotic supplementation induced significantly enhanced expression of immune- and stress-related genes, for instance, TNF-α, IL-1β, SOD, and GSH-Px in T2 and T4 groups. TNF-α enhances immune regulation by promoting lymphocyte migration to infection sites [19]. Similarly, elevated SOD and GSH-Px expression underscores the antioxidant capacity of *B. amyloliquefaciens*, mitigating oxidative injury induced by (ROS) [64]. These findings validate the dual role of Ecobiol plus^®^ in enhancing immunity and oxidative stress defenses in Nile tilapia. The histopathological analysis highlighted significant improvements in gut morphology, including increased villi length, width, and crypt depth in Ecobiol plus^®^-treated groups. These changes reflect enhanced nutrient absorption and gut health, consistent with prior studies in Nile tilapia [65]. Increased goblet cell activity contributed to pathogen exclusion and epithelial integrity, reducing the impact of *A. hydrophila*-induced damage [66].

Improvements were also observed in the gills, hepatopancreas, and spleen, with treated groups exhibiting reduced lesions and inflammation compared to controls. In the current study, the spleens of fish in the control group showed severe pathological alterations, including congestion of blood vessels, necrotic areas, depletion of white pulp, and increased melanomacrophage centers. These findings indicate significant immune suppression and tissue damage caused by the *A. hydrophila* challenge, consistent with previous studies highlighting similar stress responses in fish spleens under pathogenic attack [67].

In contrast, fish supplemented with medium and high Ecobiol plus^®^ showed substantial amelioration of splenic damage. The spleens in these groups exhibited minimal congestion, well-preserved white and red pulp, and reduced necrosis, indicating a protective effect of the dietary intervention. These improvements suggest that the probiotic treatment modulates immune responses and enhances the fish’s ability to counteract infection, which aligns with prior research demonstrating the immunomodulatory potential of Bacillus-based probiotics [67]. The observed reduction in pathological lesions supports the role of probiotics in promoting immune homeostasis by stimulating lymphocyte proliferation, reducing inflammation, and maintaining splenic architecture [68]. These findings highlight the spleen’s critical role in systemic immune function and provide robust evidence for the immunoprotective effects of Ecobiol plus^®^ in mitigating the impacts of bacterial challenges. These results reinforce the protective effects of Ecobiol plus^®^ against pathogenic and oxidative stressors.

## Conclusion

This study highlights the value of Ecobiol plus^®^ as a probiotic in the diets of freshwater fish, specifically *Oreochromis niloticus* fingerlings, by demonstrating its ability to enhance growth performance, physiological responses, and disease resistance. These findings are particularly relevant to modern intensive aquaculture practices, where stress from *A. hydrophila* is a common challenge. Ecobiol plus^®^ offers a promising alternative to antibiotics, effectively boosting the immune system of fish exposed to pathogens. Based on the significant improvements observed in growth rate and immune parameters, it is recommended to include 0.4 g/kg of Ecobiol plus^®^ in the diets of Nile tilapia fingerlings for optimal performance and health.

### The limitation of the study

The study provides valuable insights into the effects of Ecobiol plus^®^ on the growth performance, physiological response, oxidative stress, and immune response in Nile tilapia; however, it has certain limitations. The research was conducted in a controlled environment with a limited sample size, which may not fully capture variations observed in large-scale aquaculture or diverse geographical settings. The study duration was also relatively short, limiting the understanding of long-term effects. The investigation focused solely on *Aeromonas hydrophila*, leaving the efficacy of Ecobiol plus^®^ against other pathogens unexplored. Furthermore, the controlled conditions may not reflect real-world aquaculture systems, where environmental stressors like fluctuating water quality and temperature variations could influence outcomes. These factors highlight the need for further research to validate and extend the findings under varied and practical aquaculture conditions.

## Electronic supplementary material

Below is the link to the electronic supplementary material.

Supplementary Material 1

## Data Availability

The authors confirm that the data supporting the findings of this work are available within the article. Raw data that supports the findings are available upon reasonable request.
