# Supplementary material for: Impact of Ecobiol plus ® feed additive on growth performance, physiological response, oxidative status and immunological status of Nile tilapia (Oreochromis niloticus) fingerlings challenged with Aeromonas hydrophila
Source: BMC Vet Res. 2025 Jan 31;21:46. doi: 10.1186/s12917-025-04480-x (PMC11784116; doi:10.1186/s12917-025-04480-x)
Supplement: Supplementary file 1 — Supplementary Material 1 [file 12917_2025_4480_MOESM1_ESM.pdf]

Table S1: LD50 and the survival rat%

Group	LD50 Dose (CFU/mL)	Survival Rate (%)
**Control**	3×10^7	81.32
**0.1 g/kg Ecobiol plus®**	3×10^7	94.68
**0.2 g/kg Ecobiol plus®**	3×10^7	92
**0.4 g/kg Ecobiol plus®**	3×10^7	96
